# From Sufficient Health to Sufficient Responsibility

**DOI:** 10.1007/s11673-020-09992-9

**Published:** 2020-07-21

**Authors:** Ben Davies, Julian Savulescu

**Affiliations:** Uehiro Centre for Practical Ethics, University of Oxford, Suite 8, Littlegate House, St Ebbe’s Street, Oxford, OX1 1PT UK

**Keywords:** Ethics, Responsibility, Healthcare funding, Sufficiency, Sufficientarianism, Luck egalitarianism

## Abstract

The idea of using responsibility in the allocation of healthcare resources has been criticized for, among other things, too readily abandoning people who are responsible for being very badly off. One response to this problem is that while responsibility can play a role in resource allocation, it cannot do so if it will leave those who are responsible below a “sufficiency” threshold. This paper considers first whether a view can be both distinctively sufficientarian and allow responsibility to play a role even for those who will be left with very poor health. It then draws several further distinctions that may affect the application of responsibility at this level. We conclude that a more plausible version of the sufficientarian view is to allow a role for responsibility where failure to do so will leave someone else who is *not* responsible below the sufficiency threshold. However, we suggest that individuals must exhibit “sufficient responsibility” in order for this to apply, involving both a sufficient level of control and an avoidable failure to respond adequately to reasons for action.

## Introduction

Many of our choices involve associated risks to our health. We smoke, eat and drink too much, engage in risky sports and professions, or don’t do enough exercise. A recurring theme in discussions of healthcare rationing is the question of whether individuals who are appropriately responsible for their own poor health should be treated differently from those who are not.

One way to characterize this question is through a distinction between two different kinds of luck (e.g., Dworkin 1981). Bad “brute luck” covers the misfortunes we cannot control, such as being hit by lightning or contracting a childhood illness. Bad “option luck,” on the other hand, refers to unwanted things that happen as a result of our own free choices: we take a gamble, and it goes wrong. Luck egalitarians (e.g., Arneson 2006; Barry 2008; Segall 2009; Cohen 2011; Lippert-Rasmussen 2016) adopt this distinction. For instance, a standard (though not universal) luck egalitarian claim is that justice demands that people are left no worse off than others due to factors that are not in their control, that is, that we do not allow people to become worse off as a result of bad brute luck. In the realm of healthcare, this implies, *ceteris paribus*, that if one person is less healthy than others because of factors beyond their control, that demands rectification or compensation.^1^ If they are worse off because of choices they have made, however, this need not be unjust (Lippert-Rasmussen 2016, 1–25).

Consider the following two cases:

**Moderate Priority**: At the age of forty, Lynette had a highly sedentary lifestyle, despite having time to exercise during weekends. Her doctor warned her that if she did not increase her level of activity, she might suffer some related health problems. Now, at the age of fifty, Lynette has begun to suffer from lower back pain, which causes her some discomfort but does not prevent her from most daily activities. Lynette is placed on a waiting list to see a physiotherapist, along with patients like George, whose back pain is caused by a slipped disc.

**Severe Priority**: At the age of forty, Solomon was a heavy drinker; his doctor predicted that if he carried on, he would likely develop significant liver damage and might need a transplant. His doctor repeatedly offered him support in quitting, which he refused. Now, at the age of fifty, Solomon requires a liver transplant and is on a waiting list along with patients like Sunita, who needs a liver transplant due to a hepatitis B infection she contracted as a child. Each faces a significant risk of death without a transplant.

If there are no additional factors, a straightforward version of luck egalitarianism will conclude that Lynette and Solomon have no justice-based claims to treatment or at least that the priority of their claims is reduced. This is because, on the face of it, each had control over the decision to accept help changing a habit that they knew would damage their health. Many have thus accused luck egalitarians of being unduly “harsh” when it comes to abandoning the imprudent (Fleurbaey 1995; Anderson 1999; Voigt 2007). While some might see it as reasonable to make treatment for Lynette a lower priority than for George, it strikes many as overly severe to make Solomon a lower priority than Sunita.

Luck egalitarians have marshalled several responses to the charge of harshness. Some suggest that while we have no obligations of justice, we may nonetheless have reasons of compassion to treat those who are responsible for their ill health (Barry 2008, 148). Others insist that we can only hold people substantively responsible for their decisions (i.e., impose penalties on them) if they have an equal opportunity to choose well; in reality this is rarely true (Segall 2013, 178–179).

An alternative is to turn to a sufficiency principle (Fleurbaey 1995; Bou-Habib 2011; Herlitz 2019). This paper develops and refines this response and the ways sufficientarianism^2^ might accommodate considerations of responsibility. The basic thought is that we can recognize the importance of responsibility in healthcare allocation while avoiding harshness by allowing responsibility to play a role only when holding someone responsible does not bring them below a sufficiency threshold. This idea is explained, along with further details of sufficientarianism, in section II. Section III outlines an underexplored conflict in sufficientarianism: whether the view should be “responsibility-sensitive” or not. Responsibility-insensitive sufficientarians hold that even if people can be penalized for outcomes for which they are responsible, this cannot occur if doing so would leave someone below the sufficiency threshold. Responsibility-sensitive sufficientarians believe that responsibility can play some role even below the threshold.

We endorse the latter view. However, this endorsement does not exhaust the responsibility-related questions sufficientarians must answer. In section IV, we outline three further issues that responsibility-sensitive sufficientarians might disagree on and offer some answers.

Section IV.1 considers the degree of patients’ medical need. As well as being above or below the sufficiency threshold, patients within those categories can have different levels of need. We consider whether these finer-grained differences matter and conclude that, for those below a sufficiency threshold, responsibility should only apply to comparable cases of need.

We then suggest, in section IV.2, that responsibility comes in degrees and that individuals must achieve a sufficient level of control, which is more demanding than bare competence, to be held substantively responsible in a medical setting.

Finally, section IV.3 considers another element of responsibility, the quality of the reasons for which one acts. Responsibility-sensitive sufficientarianism risks punishing people for pursuing anything other than lives of extreme caution. We therefore distinguish between the ideas of reasonable and unreasonable risk, concluding that even if those who take reasonable risks which go badly are responsible for their needs, they should not be held substantively responsible in a healthcare system—that is, through different treatment compared with others.

A worry about harshness is just one of many objections to using responsibility as a rationing criterion (Sharkey and Gillam 2010; Friesen 2016). The cases of Lynette and George, and Solomon and Sunita, aim to avoid several obvious practical issues. Judgements of responsibility are difficult to make in one-off emergency situations; asking medical professionals to make them would likely benefit nobody; hence, we consider cases where responsibility is exhibited over a repeated pattern of behaviour. Moreover, we argue in section IV that it is reasonable to penalize someone for their choices only when those choices are both unreasonable and sufficiently within their control. The offer of support to both Lynette and Solomon is thus crucial to establishing the justification of responsibility.

This paper will not address all objections to the use of responsibility judgements. Rather, our aim is to show that two powerful objections to the use of responsibility can be overcome in a limited, but important, set of cases. These objections are (i) that individuals have only limited control over both their behavioural choices and the effects these choices have on their health, and (ii) that holding patients responsible for choices which worsen their health unreasonably fetishizes health, elevating it above other important goods with which it often competes in people’s lives.

In line with the first objection, we offer a proposal that focuses not on patients’ choices to adopt unhealthy behaviours but on their choices whether to accept support in taking reasonable steps to end or mitigate those behaviours.

In line with the second objection, we suggest an account of responsibility that is sensitive to whether patients’ choices are morally, epistemically, and prudentially reasonable. Only when patients’ refusal to accept support is unreasonable is it reasonable to hold them responsible.

## Sufficiency

A straightforward sufficientarian approach to responsibility is:

**Standard sufficientarian responsibility**: Responsibility may affect people’s healthcare entitlements only when this does not make them fall below, or prevent them rising above, the threshold of sufficiency.

As Fourie and Rid (2016, 2) note, many “Countries with universal access to health care are delineating ‘basic’ health care packages that all health insurance plans are obliged to cover, as opposed to services that individuals may access by purchasing voluntary, additional insurance plans or by paying out of pocket.” In other words, the concept of sufficiency is embedded in many healthcare systems. While this does not mean sufficientarianism is the correct approach to health justice, it adds to the importance of considering its applicability.

Like many philosophical terms, sufficientarianism is associated with a variety of views. In brief, sufficientarians all agree that there exists at least one threshold and that justice requires us to treat people who are below this threshold or who risk falling below it (we will call these individuals the “badly off” and say that they are in “severe need”), with *discontinuously* greater priority than those who are and will remain above it (the “well off”, who have “moderate need”).^3^ Sufficientarians do not simply think that those who are worse off have greater claims but that meeting the needs of the very badly off has a special moral urgency. However, sufficientarians disagree on several issues. Three important disagreements are:

**The level of sufficiency**: Sufficientarians all face a challenge of arbitrariness. Although they disagree on what role a threshold should play, all agree that two people can have very similar levels of some relevant good but be treated very differently because one of them is above the sufficiency threshold and the other is below (hence the idea of a “discontinuous” difference in our definition above).

More generally, sufficientarians disagree about how demanding the sufficiency threshold should be. If the threshold of sufficiency is high, standard sufficientarian responsibility implies that responsibility should play very little role in healthcare. Indeed, at some very high levels, it might imply that responsibility should play no role, since the kinds of health problems that can affect people while they remain sufficiently well off are not the concern of a public healthcare system anyway. For instance, Frankfurt (1987) suggests that sufficiency kicks in once people are “content” with what they have. But at least some people will require interventions to be content that may not be warranted under a public healthcare system, such as minor cosmetic surgery or unlimited help with fertility treatment.

Alternatively, if the threshold is low, standard sufficientarian responsibility allows responsibility to play a major role in healthcare. For instance, Shields (2016) endorses a “Principle of Sufficient Autonomy,” where people have sufficientarian claims to what is necessary for them to form beliefs about the kind of life they want to lead and to pursue it. This is consistent with significant health burdens.

**The role of sufficiency**: “Headcount” sufficientarians argue that we should maximize the number of people above the threshold (Benbaji 2005; Dorsey 2008). This view is consistent with rejecting any further claims of justice once someone has enough, but it is also consistent with the claim that once we have achieved maximization, we ought then to benefit the better off.

On the other hand, “weighted” sufficientarians (Shields 2016) argue that while the threshold has importance for claims of justice, this is because we should show special concern to those who are badly off and, importantly, a greater concern the worse off they are. To demonstrate the difference between these views, imagine that we have established a threshold of sufficiency that includes being free of all except minor pains. We then face a choice between giving painkillers to Abby, who is in moderate pain which could be removed entirely, or to Bridget, who is in severe pain which could be reduced to Abby’s moderate level. While a headcount sufficientarian will want to help Abby (since this increases the number of people above sufficiency by one), a weighted sufficientarian may prefer to help Bridget since she is worst off amongst those who are below the threshold.

Finally, weighted sufficientarians may disagree on whether to give absolute or non-absolute weight to the interests of the badly off. According to an absolute weighting, we should only be concerned with the well-off once all the badly off have been helped as much as possible. Anything less than an absolute weighting will imply that, while we should have a strong preference for the badly off, this can be outweighed by sufficiently strong claims for the well-off. For instance, if we can either provide a very minor benefit to someone who is badly off, or significant benefits to several well-off people, a non-absolute weighting might prefer the latter option. An absolute weighting must prefer the former option.

The plausibility of these various positions depends on where the sufficiency threshold is set. If the threshold is very high, it may seem more plausible to give an absolute preference to those below it. If the threshold is lower, however, such an absolute preference seems to involve abandoning a great many people who have considerable health needs.

**The currency of sufficiency**: As with any theory of justice, sufficientarians disagree about which good or goods are intrinsically important to justice. In the context of healthcare, two broad options are particularly relevant. First, sufficientarians might think health is important because of its effect on opportunity or capability and that what matters to justice is that one has sufficient opportunities. Daniels 2008 (29–78) outlines probably the most developed version of the view that health’s value is primarily related to opportunity, though with respect to equality rather than sufficiency. Shields (2016, 44–81) argues that there is a special moral importance to ensuring that people develop and retain the capacities necessary to pursue certain central opportunities, such as developing and pursuing a conception of the good. The difference in capacity between someone who can do so with considerable difficulty and someone who cannot do so at all may be minor, yet the moral difference is considerable.

Second, sufficientarians might adopt a welfarist view, where health is both instrumentally valuable to and a constitutive part of well-being. This category is broad, including hedonistic views, subjectivist views, and views where a good life consists of a plurality of irreducibly valuable “functionings” in different areas such as security, interpersonal attachments, and liberty (generally known as “capabilities” views, e.g., Sen 1979; Powers and Faden 2006; Nussbaum 2011).

These categories are not mutually exclusive. For instance, Arneson (1989) argues that the basic currency of justice is opportunity for welfare.

What is important for sufficientarianism is that whatever currency is adopted, there is at least one threshold where we have reason to treat people who occupy different sides of that threshold substantially differently, even if their positions are only moderately different.

Our approach is as follows. We consider a sufficientarian approach which is pluralist in currency, sets low thresholds related to these currencies (e.g., the avoidance of severe pain and the retention of capacities necessary to form and pursue plans), and places a strong but non-absolute weight on achieving and maintaining sufficiency. As we have suggested, placing non-absolute weight on sufficiency makes it more plausible to set a low threshold: since a concern with sufficiency is not absolute, such an approach does not ignore significant health needs above the sufficiency threshold. This approach also recognizes that healthcare has multiple goals which cannot necessarily be reduced to a single metric.

## Sufficiency: Responsibility-Sensitive or Not?

Many existing discussions of sufficiency and responsibility outline the idea that a sufficientarian threshold should represent the point at which responsibility no longer applies, operating a “responsibility-insensitive” (Gosseries 2012) approach below the threshold. Fleurbaey (1995, 40) notes that responsibility-sensitive allocation procedures will see no problem in leaving someone who makes “the slightest mistake that [puts them] in a hazardous situation,” suggesting that we must balance responsibility against the scale of disadvantage suffered. Bou-Habib (2011, 299) endorses a view that would give each individual equal access to resources across their lives but suggests that these shares must be distributed across lives in a way “that either (a) we can always endorse our life plans, or (b) we always have a decent set of opportunities at each stage of our lives.”

An important exception is Herlitz (2019, 936), who argues that while a sufficiency principle is essential to understanding our obligations towards those who are responsible for their own poor health,

“there are limits to the obligations we have toward prioritizing the worse off who are where they are fully due to faults and choices of their own. There might … be an important difference between the obligations we have to the group who are badly off due to no fault or choice of their own and to individuals who have themselves to blame for their misfortunes.^4^

While the responsibility-insensitive position is that responsibility can never play a role when people are, or will be, left badly off, Herlitz notes that we sometimes face conflicts *between* people who do not have enough, some of whom are responsible for their position and some of whom are not. If we cannot help everyone, some people must be left badly off.

It is worth considering the distinction employed here in relation to our cases outlined above. Both positions will likely endorse treating George before Lynette in Moderate Priority, since while this may cause some suffering for Lynette, her suffering will likely not be severe, nor will her central capacities for pursuing central opportunities be threatened.

The positions will differ, however, when it comes to Severe Priority. A responsibility-insensitive position will note that although Solomon is responsible for his poor health, he is at risk of becoming badly off. Since both he and Sunita risk becoming badly off if not treated quickly, we should use some other way to decide between them. A responsibility-sensitive view like Herlitz’s will insist that Solomon’s responsibility for his health needs comes with a cost even if he stands to become very badly off. Since Sunita faces the same risk, an appeal to sufficiency cannot justify ignoring Solomon’s responsibility.

## Developing Responsibility-Sensitive Sufficiency

We also believe that responsibility sometimes has a role to play even when those who are responsible will likely fall below the threshold of sufficiency. Both Solomon and Sunita have a claim to have their lives saved. Whereas responsibility can invalidate one’s claim to aid when one will be left only in moderate need, it can only ever weaken it when one will be left in severe need. Nonetheless, if only one can be saved, Sunita’s claim is greater. However, there are several further issues to consider, which the adoption of responsibility-sensitive sufficientarianism does not resolve.

### Degrees of Need

The first of these issues is the relationship between medical need and responsibility below the threshold. One possible view (which seems to be Herlitz’s) is that there are two categories of responsibility (responsible / not responsible) and two categories of medical need in reference to the sufficiency threshold (severe / moderate). Herlitz’s view appears to be that those who are responsible for their own severe need should be prioritized over those who are not responsible but who have only moderate need; however, those who are responsible for severe need should take a lower priority compared with those who have a severe need and are not responsible.

But medical need comes in degrees. To take the example of pain, even if one agrees that there is something morally distinctive about severe pain, there can be more and less severe cases within this category. Similarly, among those individuals whose medical conditions prevent them from forming and pursuing their own conception of a good life, there are important variations in mental and physical capacity. There are also differences in risk. In Severe Priority, we described Solomon and Sunita as both facing a “significant risk” of death. But that is consistent with Solomon facing a *much higher* risk than Sunita.

One possibility is that those who are responsible for their severe need should always be a lower priority when competing with those who are not responsible for their severe need, no matter how great a gap there is between their positions.

As we note in section II, sufficientarians have disagreed about what the precise moral role of the threshold should be. However, many argue that when deciding among individuals who are below the threshold, we should engage in something like prioritarian reasoning, where benefits to the worst-off matter more. To recap, this would mean that if we had to confer a benefit of equal size, we should prefer to give it to the worse-off individual. However, in cases where the benefit we can offer to the worst-off is significantly smaller than that which we can offer to the better off, we may prefer the better-off.

This view lends itself to a parallel claim about responsibility. Consider again our case of Severe Priority. Even though both Solomon and Sunita are in severe need following their accident, the degree of need may nonetheless be relevant to whether and how to involve responsibility. For instance, imagine that Sunita faces 5 per cent of dying if not treated within the next month, whereas Solomon is in a critical condition, with a 90 per cent chance of dying if not treated immediately.

Although Solomon is responsible for his condition, recall that the responsibility-sensitive sufficientarian view is that his claim is *weakened,* not eliminated. It is plausible that, in an ordinary case where neither patient is responsible, the patient with significantly more severe need has a stronger claim to priority. This means that if there is a considerable difference in the severity of need in Solomon and Sunita’s case, it may still be right to prioritize Solomon. Although his claim to treatment is weakened by his responsibility, it was significantly greater to begin with. On the other hand, if Solomon and Sunita were in a comparable state, or if Solomon’s need was much less severe, it would be right to prioritize Sunita.

Compare this with the Moderate Priority case of George and Lynette. Since Lynette will not be left badly off, we view her claim as *undermined* by her responsibility. As such, so long as Lynette will not be left badly off, it is right to prioritize George’s care over hers, even if she is significantly worse off than he is.

### Degrees of Control

We have noted that medical need comes in degrees and claimed that this is relevant for responsibility-sensitive sufficientarianism. According to some views, it is also true that responsibility comes in degrees. For instance, Coates and Swenson argue that one can be more or less responsible depending on how well one is able to recognize and respond to reasons for action, both capacities which can vary by degrees. As Coates and Swenson suggest, one’s capacities for reasons recognition and response can be significantly affected without being entirely debilitated to the point where one lacks responsibility altogether. They discuss a case where a person’s severe depression means that she fails to pick up a friend from the airport, even though it is true that she would have left her house in response to other reasons, such as a fire. That she is still somewhat reasons-responsive shows that she still has moral responsibility. Yet that she faces a significant barrier to responding to reasons should, according to Coates and Swenson (2013, 634), “mitigate her responsibility and blameworthiness.” Nelkin (2016) adds that one’s degree of responsibility can vary depending on how reasonable it is to expect a person to take the opportunities with which they are presented. And Tierney (2019) suggests that as well as being affected by how *difficult* a person finds a particular choice, the degree of responsibility they face depends on the quality of the reasons for which they act, an idea we develop in the following section.

If moral responsibility comes in degrees, this raises a further question for responsibility-sensitive sufficientarianism: should all people who are responsible for their ill health be treated alike (within the categories of the well-off and badly off)?

We can defend a scalar conception of responsibility but acknowledge that it is also a threshold concept. For instance, just as people’s reasons-responsive capacities can affect *how* responsible they are, at some point a person can be so unresponsive to reasons that it is inappropriate to hold them responsible at all. In our view, this is because an individual’s inability to respond to reasons effectively undermines their ability to rationally control their choices in a reasonable way—that is, their autonomy. However, a lack of autonomy is not the only way in which someone might fail to have a sufficient degree of control. As well as lacking sufficient internal capacities to exercise proper control, someone might also lack sufficient opportunities because of their external circumstances. An insufficient availability of good choices, or excessive burdens being attached to those options, also undermine an individual’s ability to sufficiently control their health. It is thus inappropriate to hold them substantively responsible for choices which damage their health.

An alternative view (e.g., Roemer 1993; Segall 2013, 179) is that responsibility can only be relevant to distributive decisions when people have *equal opportunity* to choose well. The fact that someone chooses a poor diet may look like an exercise of responsibility, and in a narrow sense it is. But this person's choices may be heavily influenced by having lived in constrained circumstances while growing up, which both influenced her tastes and deprived her of adequate nutritional education. In addition, she may currently lack adequate time, money, or energy to research, buy, and cook nutritious meals. So, while she does not exactly *lack* the relevant capacities (she could in theory eat better), she cannot be held substantively responsible because she has not had equal opportunities.

However, it is not clear why people must have equal, as opposed to merely sufficient, opportunities to make good choices. Consider again a person who eats in ways that do not provide them with adequate nutrition. We have seen already that one constraint may be time: Elaine, who must work two demanding jobs to get by, lacks the time to cook. But the problem is not that she has *less* time than, say, a billionaire, Elon, who hires a professional chef and nutritionist. Carl, who has a secure, well-paid job also does not have as much time as Elon does to ensure that his meals are adequately nutritious, and so Carl faces an inequality of sorts compared with Elon. Nonetheless, Carl does not suffer in the same way as Elaine, who has barely any time for personal care. While Carl has less time than some, he still has enough time to cook adequately nutritious food. He could thus be fairly held responsible for choosing not to do so.

One might worry that this approach will tolerate intuitively objectionable disparities. Rather than comparing someone in secure, well-paid employment with a millionaire, we might instead compare them with a person who has *barely adequate* time to prepare food. Similar points arise for other kinds of health-promoting opportunity. Significant health inequalities seem likely to emerge. Does our analysis imply, counter-intuitively, that these inequalities do not matter because everyone involved has sufficient opportunity to promote their health?

Our answer involves pushing back on precisely what “barely adequate” opportunity means in this context. On reading this phrase, one might naturally imagine a person who struggles greatly to maintain good health yet does manage to do so. After all, one might think, if they *successfully achieve* good health, they must have had an adequate opportunity, even if it is barely adequate.

We resist this interpretation. The idea of sufficient responsibility, while a threshold concept, is not equivalent to the point at which we may begin to hold people responsible in any sense. The problem with Elaine’s situation is not that she is so constrained as to be entirely free of responsibility but that her degree of responsibility is insufficient to warrant being penalized for her choices. The degree of control which she has over her situation is considerably constrained by other moral obligations, financial needs, and knowledge. She is more responsible for her diet than others are; she may also be sufficiently responsible for certain types of judgement.^5^ Yet she is not sufficiently responsible to be held *substantively* responsible, in the form of penalties including the expression of blame, for health problems she may incur.

Moreover, the fact that a person achieves good health does not mean that they have adequate opportunity to do so, since this does not take into account what else that individual had to sacrifice. As we outline in section IV.3, there are often good moral, epistemic, and prudential reasons to sacrifice one’s health to some extent. That a person can find the time and money to cook nutritious food does not entail that one has adequate opportunity to do this, since doing so might involve *unreasonable* sacrifices elsewhere.

Secondly, however, we suggest that even while a sufficientarian approach requires a threshold concept of control, this is compatible with one’s degree of control also making some difference. Crucially, what one’s degree of control makes a difference to is how reasonable it is for you to make particular choices. We regard both Elon and Carl as having adequate time to eat nutritiously; it may nonetheless be true that Carl is *more likely* than Elon to face situations where it is reasonable for him to sacrifice his health and thus avoid substantive responsibility for resulting health problems. The same is not true for those who fail to have sufficient control, since the idea of sufficient control is that one must be above the threshold to be held substantively responsible. Two people who fail to have sufficient control may have different degrees of control, but this does not mean they should be treated differently from a substantive perspective.

It is difficult to be precise about the level at which one has sufficient control so as to be legitimately held substantively responsible. Nonetheless, we can establish some apparently clear cases. Again, compare the case of the working parent with one of our original cases, Severe Priority. Let us fill in some more details and stipulate that Solomon is fully aware of the dangers of drinking and decides to take the risk simply because he is happy to take chances with his future welfare in order to increase his satisfaction in the present. Clearly, Solomon had ample opportunity to make the right decision, both in terms of internal capacities and external means. As such, it is reasonable to hold him substantively responsible for his decision. Nonetheless, given the dangers of holding a person responsible when they *lack* sufficient opportunity, the range of cases to which responsibility can be applied as a distributive criterion are, as we discuss in more detail further on, limited to those where patients have refused a clear and accessible opportunity of support to change an unhealthy habit.

However, the fact that the degree of control a person can exercise affects their degree of responsibility and hence the appropriateness of holding them substantively responsible in healthcare does raise a further issue for our initial cases. While we have hypothetically filled in the details of Solomon’s decision to make it clear that he is sufficiently responsible, in the real world this information is unlikely to be readily available. As such, even if it is theoretically permissible to hold people substantively responsible in cases like Moderate Priority and Severe Priority , we should exercise a considerable degree of caution in such cases and a greater degree of caution when patients are at risk of becoming badly off—that is, of falling below the sufficiency threshold.

It may be, then, that in practice, responsibility should play a role only where we can have reasonable belief that patients have sufficient control over their behaviour. One way to ensure this is to look to cases of *repeated* irresponsibility. Consider an ideal instance of exercising health-related responsibility, where a fully autonomous patient is deliberately and carefully offered a reasonable chance to change behaviour that is causing, exacerbating, or threatening a health burden (Savulescu 2018). Now imagine that a person is repeatedly offered such opportunities and repeatedly fails to take them and so must repeatedly be the subject of costly interventions. Such an individual shows such disregard for the burdens that their care places on others that at some point we can allow their failure to exercise the responsibility of which they are capable to influence their claim to further care. What’s more, the establishment of a pattern of behaviour allows for a reasonable judgement about whether a patient has sufficient control over their behaviour.

It is worth noting that this argument does not require that we abandon the repeatedly imprudent altogether. Rather, it may be that responsibility’s role is in moving people from a first-choice treatment to a less attractive treatment or a longer waiting time. For instance, someone who responds to successive liver transplants by continuing the drinking habit that gave them cirrhosis in the first place may no longer qualify for future transplants, have to wait longer, or only qualify as a candidate if there are no other suitable candidates. But they will still qualify for palliative care. Alternatively, repeated irresponsibility may mark a point at which additional conditions may be legitimately placed on care, such as counselling for alcohol abuse or even taking Naltrexone or Disulfiram, both of which are used as part of treatment for alcoholism. The key point is that a responsibility-sensitive sufficientarian view need not commit us to treat this patient as we would someone who incurred multiple health problems in ways that were not due to the exercise of her responsibility.

### Reasonable Risk

A third issue concerns the quality of the reasons for which a person acts. We have already mentioned Tierney’s view that the degree of responsibility a person is subject to depends in part on the quality of their reasons. Tierney mentions two ways in which the quality of a person’s reasons can vary. Reasons can have different *moral* qualities. For instance, failing to keep to an appointment because you are comforting a friend is less serious than failing to keep an appointment because your favourite film is on TV. Reasons can also have different epistemic qualities. Failing to pick up a friend because you dreamt their flight was cancelled is worse than failing to do so because of misleading information on the airline’s website.

In our view, whether a person’s responsibility should be allowed to impact their condition depends on whether the risk that they take is reasonable. The moral and epistemic qualities of reasons that Tierney raises form two of the three planks that comprise the reasonableness of a risk. The third plank is the prudential quality of a person’s reasons.

An individual’s health is of clear prudential value to them. Health is both constitutively and instrumentally good for you; illness is constitutively and instrumentally bad for you. However, the *pursuit* of health has opportunity costs: always choosing what will improve your health may reduce your ability to enjoy other things that are good for you. Since health is not the only component of well-being, it is often prudentially reasonable to choose the less healthy of several options when that will improve your well-being overall.

This basic idea can help to explain the common view that it is unfair to penalize people for certain types of choices. For instance, it is unreasonable to penalize someone for eating unhealthily when almost all of their realizable food options are unhealthy. If fresh food is not readily available, accessing it may require an expenditure of time or money that individuals do not have. The decision to eat unhealthily in such a context is therefore prudentially entirely reasonable and thus not one for which anyone could be reasonably penalized.

Similar considerations apply to the moral and epistemic qualities of one’s reasons. A person’s actions might be unreasonable not only because the value of his goal does not prudentially justify the level of risk he puts himself under but also because it does not *morally* justify the level of risk he puts others under. Solomon and Lynette’s decisions are morally neutral in themselves. But people can sometimes have excellent moral reasons for sacrificing their health. A person who chooses to feed her children rather than herself makes a choice that harms her health. But the quality of her reasons (her wish to care for her children) shows that this is a morally reasonable risk to take.

A person’s pursuit of a worthy goal might be epistemically unreasonably reckless because they have formed relevant beliefs (e.g., about the worthiness of the goal or the efficacy of their chosen means) in epistemically unjustified ways. Solomon could not, for instance, appeal to a belief, even if it were sincere, that significant alcohol consumption would not affect him as it does other people. Given the evidence he has, including warnings from a medical professional, this is not a reasonable belief to hold. Compare this to a person who smoked before it was widely known that smoking caused lung cancer; their belief that they were doing something harmless—perhaps even healthy—is epistemically reasonable.

Attaching penalties to risk without making these distinctions is itself risky. It risks constraining the kinds of good at which people aim in their lives (Savulescu 2002). Taking risks can lead to harm, but it can also lead to much greater good than a life lived entirely cautiously. It is therefore a disadvantage of a healthcare policy if it punishes people for taking reasonable risks. Responsibility-sensitive sufficientarians should therefore, all else being equal, prioritize those who end up badly off due to reasonable risks over those who end up badly off due to unreasonable risks.

The application of the idea of reasonableness to responsibility and justice is not new. The most developed example is Shlomi Segall’s (2009, 20) claim that we should simply define “brute luck” as, roughly, outcomes that “it would have been unreasonable to expect an agent to avoid.” Our notion of reasonable risk is close but different to Segall’s. As Segall says, his own view “shifts the focus of attention from the individual to the society,” because it “asks not whether the individual has acted in a reasonable way, but rather whether it is unreasonable for society to expect the individual to avoid a certain course of action.” Our interest remains in the question of whether the individual has acted with a sufficient degree of reasonableness by showing sufficient care for the quality of the reasons for which they act.

Consider a non-health-related example of Segall’s (2009, 23–24): in his view, it would not be unreasonable for society to expect me to refrain from giving half my money to charity, even though I might do so for morally excellent reasons.^6^ Since his is an egalitarian rather than a sufficientarian view, he suggests that a focus on the quality of an agent’s reasons would generate the absurd conclusion that we should compensate them for their voluntary loss of salary. A focus on what it is reasonable for society to expect does not.

Since ours is a sufficientarian view, it does not have the absurd conclusion that concerns Segall. To translate the example to one relevant to health, if the loss of half my salary resulted in slightly worse health (because I had to, say, give up my personal dietician and trainer), that is of only very weak sufficientarian concern. So even if the purported moral reasonableness of this decision means that it does not qualify as something for which I am responsible, all that would mean is that I should not be discriminated against when competing with other patients with similar, minor reductions in health.

## Conclusion

We have outlined a novel sufficientarian approach to the issue of responsibility in healthcare, raising and offering tentative answers to some important questions that responsibility-sensitive sufficientarians must face. While we depart from the standard sufficientarian view in allowing judgements of responsibility to play a role in healthcare allocations even for those who are badly off, we have also outlined important conditions of “sufficient responsibility” which heavily constrain the use of responsibility judgements. This paper has thus not overturned the considerable arguments against using judgements of responsibility comprehensively throughout a healthcare system. Rather, we have suggested that in some, narrow circumstances, judgements of responsibility may properly play a role in resource allocation.

## Footnotes

1 Strictly, most versions of luck egalitarianism are comprehensive, rather than applying in discrete spheres such as health. As Preda (2018) argues, this most plausibly means that entitlements to having one’s health improved depend not on whether one has worse *health* than others but on whether one is worse off than others all things considered. For simplicity, we assume in this paper that health inequalities track broader inequalities. See also Gosseries (2016).

2 While we focus on sufficientarian responses, it is worth noting that some egalitarians also respond to this issue by invoking sufficiency (e.g., Segall 2009). The central difference is that sufficientarians see sufficiency as a demand of justice, while what we might call “sufficiency-constrained” views, such as Segall’s egalitarianism, see it as a demand of broader morality outside of justice.

3 This includes the belief that those above the threshold have no justice-based claims at all.

4 See also Fabre (2007, 37), though this view is not developed in detail.

5 For instance, her doctor should advise *her* on ways to improve her diet, rather than advising someone else how to improve her diet.

6 In our view this example supports our interpretation of reasonableness. Segall suggests that it would be reasonable for a society to expect me *not to give* all my money to charity. But it is more plausible to think that others might reasonably refuse to *bear the costs* of that choice on the grounds that my decision was not prudentially reasonable.
